# Antibacterial Properties of Alkaloid Extracts from* Callistemon citrinus* and* Vernonia adoensis* against* Staphylococcus aureus* and* Pseudomonas aeruginosa*

**DOI:** 10.1155/2016/6304163

**Published:** 2016-01-20

**Authors:** Donald Mabhiza, Tariro Chitemerere, Stanley Mukanganyama

**Affiliations:** ^1^School of Pharmacy, College of Health Sciences, University of Zimbabwe, Mt. Pleasant, Harare, Zimbabwe; ^2^Department of Biochemistry, University of Zimbabwe, Mt. Pleasant, Harare, Zimbabwe

## Abstract

The development of new antibiotics from new chemical entities is becoming more and more expensive, time-consuming, and compounded by emerging strains that are drug resistant. Alkaloids are plant secondary metabolites which have been shown to have potent pharmacological activities. The effect of alkaloids from* Callistemon citrinus* and* Vernonia adoensis* leaves on bacterial growth and efflux pump activity was evaluated on* Staphylococcus aureus* and* Pseudomonas aeruginosa*. At a concentration of 1.67 mg/mL, the alkaloids inhibited bacterial growth with comparable effects to ampicillin, a standard antibiotic. The alkaloids from* C. citrinus* were the most potent against* S. aureus* with an MIC of 0.0025 mg/mL and MBC of 0.835 mg/mL. It was shown that effects on* P. aeruginosa* by both plant alkaloids were bacteriostatic.* P. aeruginosa* was most susceptible to drug efflux pump inhibition by* C. citrinus* alkaloids which caused an accumulation of Rhodamine 6G of 121% compared to the control. Thus,* C. citrinus* alkaloids showed antibacterial activity as well as inhibiting ATP-dependent transport of compounds across the cell membrane. These alkaloids may serve as potential courses of compounds that can act as lead compounds for the development of plant-based antibacterials and/or their adjunct compounds.

## 1. Introduction

Bacterial infections are one of the leading causes of infectious diseases in Africa. Poverty and poor health infrastructure continue to be an impediment to effective health care service delivery [1]. On a global scale, life-threatening infections caused by these pathogenic prokaryotic microorganisms have become an important cause of morbidity and mortality in immune-compromised patients in developing countries [2]. Despite the availability of a wide range of antibiotics, bacteria are constantly developing resistance to these agents [3], which makes it difficult for the concerted effort of combating infectious diseases. From the advent of antimicrobial application in treatment of bacterial diseases, bacteria responded by manifesting varied forms of mechanisms of resistance. With passage of time the level and complexity of the resistance mechanisms by bacterial pathogens increase [4]. Drug resistant bacteria, particularly* Staphylococci* species,* Klebsiella pneumoniae,* and* Pseudomonas* species, are becoming commonplace in healthcare institutions [4] and are possibly contributing to the occurrence of treatment failures.

Virulence factors aid bacteria in invading tissues, evade the defenses mechanisms, and cause disease in the host [5]. It is fundamental to understand how pathogenic bacteria interact with their hosts to produce disease as these mechanisms may be targets in drug development [6]. The ability to form biofilms confers a selected advantage for bacteria to strive under harsh environmental conditions and provide resistance to antimicrobial agents [6].* Pseudomonas aeruginosa*,* Mycobacterium tuberculosis*,* Streptococcus pneumoniae,* and* Staphylococcus aureus* are examples of bacteria that form biofilms [7].

*Staphylococcus aureus* and* Pseudomonas aeruginosa* are typical Gram-positive and Gram-negative pathogens, respectively, that have been significantly characterized in community-acquired and hospital-acquired infections [8].* Staphylococcus aureus* is a facultative anaerobe that exists normally as part of the skin and nasal flora and estimates are that 20% percent of the human population are long term carriers of the bacteria [9]. This bacterium also occurs in animals, sewage, and food and on household and environmental surfaces. The presence of* S. aureus* in humans as part of normal flora means that the bacteria can infect human tissues such as the skin dermis or mucosal linings when defensive barriers have been breached. This leads to topical skin infections like furuncles, carbuncles, acne, boils, and severe staphylococcal-scalded skin syndrome [10]. Life-threatening systemic infections such as pneumonia, meningitis, and sepsis may also result especially in immunocompromised patients [11].* P. aeruginosa* is a ubiquitous microbe as it can catabolise a wide range of organic chemicals like benzoate; as such, it has been found in environments such as soil, water, and hospitals and in humans, plants, and sewage [12]. An outer membrane in the cell structure of* P. aeruginosa* confers pronounced resistance to xenobiotics including antibacterials.* P. aeruginosa* rarely causes diseases in health individuals with a competent immune set-up; it, however, is an opportunistic pathogen that infects immunocompromised patients, especially those with AIDS and cystic fibrosis and chemotherapy patients [13].* P. aeruginosa* gains entry to burns, breached skin, or mucosal linings using its flagellum and pili and replicates to create an infectious critical mass. More seriously, the exotoxins and endotoxins released by* P. aeruginosa* continue to cause inflammation and harm even after the bacteremia has been treated with antibiotic, which makes infections by* P. aeruginosa* life-threatening [14].

One of the mechanisms of manifesting resistance to antibacterial agents is the acquisition of efflux pumps that extrude the antibacterial agent from the cell before it can reach its target site of action [4]. However, multidrug resistance is usually a combination of resistance mechanisms. The action of efflux pumps is specially noted among* P. aeruginosa* clinical strains. The efflux pumps prevent accumulation of drugs within the bacterium from achieving bactericidal or bacteriostatic concentrations at the target sites. The efflux pumps often work in synergism with limited permeability of the* P. aeruginosa* outer membrane to produce resistance to *β*-lactams, fluoroquinolones, tetracycline, chloramphenicol, macrolides, and aminoglycosides [15]. Analysis of* S. aureus* isolates that were resistant to antibacterial agents and were causing nosocomial infections revealed that these strains contained plasmids coding for transporters. These resistance mechanisms have been attributed to the occurrence of strains like methicillin resistant* Staphylococcus aureus* (MRSA) which is resistant to methicillin, tetracycline, chloramphenicol, and aminoglycosides [16]. MRSA is a major source of hospital-acquired infections and old antibiotics like vancomycin are now being used for treatment of MRSA infections despite their unfavorable side effects. The incidence of MRSA continues to increase globally and poses enormous therapeutic problems [17].

The worldwide escalation in both community- and hospital-acquired antimicrobial bacteria is threatening the effective treatment of patients, emphasizing the need for continued surveillance, prudent infection control, and new treatment alternatives [17]. Antibiotic resistant bacteria are increasingly prevalent and new antimicrobials are needed to control these pathogens yet development of resistance is unavoidable as it is a pivotal aspect of microbial evolution [1]. Thus, there is an urgent need for development of novel antibacterial products that act on molecular targets that act against bacterial resistance mechanisms.

Herbal medicine use employed either in traditional medicine practice or complementary and alternative medicine (CAM) is popular for 80% percent of the world in Asia, Latin America, and Africa and is reported to have minimal side effects [18]. Furthermore, with concerns of rising costs of drug development, plants have turned out to be some of the most cost-effective and most affordable alternative sources of drugs [19].* Vernonia adoensis is* an herbaceous plant of the Asteraceae family, which is indigenous and commonly distributed throughout Zimbabwe, where it is locally known in vernacular* Shona* language as* Musikavakadzi* [20]. Its habitat is commonly* Miombo* woodlands and wooded grassland especially near streams [20, 21]. This plant has been traditionally used in African ethnomedicine for the treatment of fever and upper respiratory tract infections and currently for TB in traditional medicine practice [22].* Callistemon citrinus*, commonly known as Bottlebrush, is a woody shrub widely distributed in the temperate regions, notably Australia particularly on the east and southwest coasts, South America, and tropical Asia, and, thus, is exotic to Zimbabwe [23]. The different parts of this herb have been used in folk medicine as a common remedy for treatment of diarrhea, dysentery, rheumatism, and antibronchitis [23].

Herbal preparations are now readily available as over-the-counter products with numerous medical claims, some of whose scientific proof remains lacking. It is, therefore, essential that, as herbal medicine use is increasingly becoming popular, scientific research is fundamental to back up such uses and medical claims and allay fears of potential herb-drug interactions with conventional medicines. Promising results from work on crude extracts have shown the potential for antibacterial activity from these two plants [24] and, hence, the need to evaluate the alkaloid extracts with a view to understanding the mechanisms of action needed to thwart the effect of both hospital- and community-acquired multidrug resistant organisms.

The main objective was to evaluate alkaloid extracts isolated from* C. citrinus* and* V. adoensis* for activity against* S. aureus* and* P. aeruginosa.*

## 2. Materials and Methods

### 2.1. Plant Material and Preparation of Extracts

Plants were collected from Mashonaland Central for* Vernonia adoensis* (Centenary: 16.8000° S, 31.1167° E), and Harare for* Callistemon citrinus* (University of Zimbabwe: 17.7840° S, 31.0530° E) on the basis of indigenous knowledge uses. The leaf samples C1 E7 (*Vernonia adoensis*) and UZ2 E7 (*Callistemon citrinus*) were authenticated by a taxonomist, Mr. Christopher Chapano of the National Herbarium and Botanic Gardens, Harare, Zimbabwe. Preparation of plant extracts was as described by Chitemerere and Mukanganyama [25]. Leaves of* Callistemon citrinus* and* Vernonia adoensis* were separately predried in a Labcon orbital incubator (Labotec Co., Cape Town, S. A.) at 50°C. The leaves were then separately ground in a two-speed blender (BL2, ABB, Moulinex, France). Approximately 5 g of* Callistemon citrinus* and* Vernonia adoensis* comminuted leaf samples were mixed with 15 mL and 20 mL of 10% v/v ammonia solution, respectively. Extraction was done with 30 mL and 50 mL ethanol for* Callistemon citrinus* and* Vernonia adoensis* samples, respectively, in a 40°C water bath for 10 minutes. A Whatman filter paper number 1 was used for filtration into separately labeled 50 mL falcon tubes. Ethanol was left to evaporate in an incubator at 50°C for 48 hours. A constant dry weight of each plant alkaloidal extract was obtained.

### 2.2. Microorganisms, Growth Conditions, Resuscitation, and Standardization

A 1 L broth medium was prepared by dissolving 15 g tryptone, 3 g yeast, 6 g sodium, and 1 g glucose in 1 L of hot distilled water and poured in a 1 L glass jar. The broth medium was sterilised in an autoclave machine (Accu Steriliser, VWR Scientific products Co., USA).* Staphylococcus aureus* (ATCC 9144) and* Pseudomonas aeruginosa* (ATCC 27853) were obtained from the Division of Microbiology, Department of Biological Sciences at the University of Botswana. The strains were maintained as stock strains in 50% glycerol in Eppendorf microtubes and kept at −33°C until resuscitation. Approximately 20 mL of the broth was inoculated with 25 *μ*L of* Staphylococcus aureus* and* Pseudomonas aeruginosa* each in its own falcon tube. The inoculated bacteria were incubated for 24 hours at 37°C in a Lab-Companion incubator (SI300 Incubated shaker, Jeiotech, Korea). Standardization of the bacterial cultures was done and bacterial broth cultures of 1 × 10^6^ cfu/mL for both* P. aeruginosa* and* S. aureus* were made.

### 2.3. Antibacterial Susceptibility Tests

The alkaloid extracts of* Callistemon citrinus* and* Vernonia adoensis* made up to a concentration of 25 mg/mL were separately applied to 96-well plates in 20 *μ*L volumes so as to expose the test cell cultures with 500 *μ*g (1.67 mg/mL) of extracts in each well.* Staphylococcus aureus* and* Pseudomonas aeruginosa* at a concentration of 1 × 10^6^ cfu/mL and broth media were diluted into the 96-well microplate wells. Each well was made to contain a total of 300 *μ*L. A positive control containing ampicillin was also set up in which 500 *μ*g was applied in each well containing media and bacterial broth culture. Relevant negative controls containing the media only or the media with extract were also set up. Preincubation absorption measurements were determined using a microplate reader (SpectraMax Plus384, Molecular Devices Co. USA) at 600 nm. Postincubation cell density measurements were also determined after incubation at 37°C in a Lab-Companion incubator for 24 h (SI300 Incubated shaker, Jeiotech Co., Korea).

### 2.4. Minimum Inhibitory and Minimum Bactericidal Concentration Determination

MIC and MBCs were also determined for the alkaloids. The alkaloid extracts were then separately serially diluted twofold from 1.67 mg/mL to 0.0032 mg/mL in a 96-well polystyrene microplate to obtain 10 dilution concentrations. In a separate 96-well microplate, 20 *μ*L of each dilution was sequentially added in triplicate into wells. Broth cultures of* S. aureus* and* P. aeruginosa* (1 × 10^6^ cfu/mL) were then separately added to the wells in 100 *μ*L volumes, each species in its own microplate after which 180 *μ*L broth media were added to the well to obtain a total volume of 300 *μ*L in each well. Rows of wells with broth media containing 100 *μ*L of cell cultures were used as positive control; also controls of media containing extract only and extract-free broth media were also used as negative controls in each well. Preincubation absorbance values were read from a microplate reader (SpectraMax Plus384 Absorbance Microplate Reader, Molecular Devices Co., USA). The microplates were then incubated at 37°C in a Lab-Companion incubator (SI300 Incubated shaker, Jeiotech Co., Korea) for 24 h; thereafter, absorbance values were read and recorded. MTT was used to identify the MIC values in the well where the purple colouration was the least visible in each test row. The experiment was performed in triplicate.

### 2.5. Drug Efflux and Accumulation Using Rhodamine 6G

*S. aureus and P. aeruginosa* were grown in two separate culture flasks overnight at 37°C with shaking at 120 rpm in a an incubator. The bacterial cultures were then poured into 50 mL centrifuge tubes and centrifuged using a centrifuge machine (MSE Minor 35, MSE Ltd., England) at 3 000 rpm for ten minutes and the supernatant was discarded. The pellet was washed twice with phosphate buffered saline (PBS) and resuspended in the buffer. The resuspended cells were poured in preweighed centrifuge tubes and spun again at 4 000 rpm for 5 minutes. The supernatant was decanted and the cells were washed again in PBS, before being centrifuged again at 4 000 rpm for 5 minutes. The supernatant was decanted and the pellet was weighed. The pellets, both of* S. aureus* and* P. aeruginosa,* were suspended at 40 mg/mL in PBS containing 10 mM sodium azide (NaN_3_). Rhodamine 6G was immediately added to a final concentration of 10 *μ*M and incubated the cells with 90 rpm agitation for 1 h in a Lab-Companion incubator (SI300 Incubated shaker, Jeiotech Co., Korea). The tubes were then divided into two centrifuge tubes in the ratio 1 : 3, to give tube A and tube B of each of the two species.

The tubes were then centrifuged at 4 000 rpm for 5 minutes Centronic SP Selecta centrifuge (Barcelona, Spain). The supernatant was discarded and cells from tube A of each of the two bacterial species were resuspended in PBS alone at 40 mg/mL. Cells from tube B of each of the two species were resuspended in PBS containing 1 M glucose. The cells of tube B of both bacteria were then divided into 5 mL portions in 8 different falcon tubes; of them, two tubes separately for glucose only, glucose + reserpine, glucose +* C. citrinus alkaloid extracts*, glucose +* V. adoensis* alkaloid extracts, and cells from tube A were divided into two 5 mL portions. This was done for each of the pellets from* S. aureus* and* P. aeruginosa*. To tubes marked glucose + reserpine, reserpine was added to a final concentration of 61 *μ*g/mL and the alkaloids added to their respective tubes to a final concentration of 61 *μ*g/mL. The centrifuge tubes were mixed by shaking on a Vortex mixer (Thermolyne Maxi Mix II, IOWA, USA) and placed in a shaking incubator (SI300 Lab-companion Co., Korea) at 37°C for 30 minutes at 90 rpm. After 30 minutes, the tubes were centrifuged at 4 000 rpm for 10 minutes in a Rotofix 32 centrifuge (Hettich, Zentrifugen, Germany) and the supernatant was collected to quantitate the amount R6G pumped out from the cell. The pellet in each tube was lysed by resuspending in 5 mL 3 M glycine pH 3. The tubes were mixed by shaking on a vortex mixer and then incubated with shaking for 24 h at 37°C. The mixture was centrifuged at 4 000 rpm for 10 minutes and the supernatant was collected and quantified for the amount of R6G that was accumulated in the cells. R6G was determined from the samples using a standard R6G calibration curve after determining the absorbance in 96-well microplates, at 527 nm, using a microplate reader (SpectraMax Plus384, Molecular Devices Co., USA).

### 2.6. Statistical Analysis

Data analyses were performed using GraphPad Instat software (GraphPad Prism Inc., San Diego, CA, USA). Levels of significance were determined using ANOVA using Dunnett's posttest where all columns of treatments were compared to the control. All data were expressed as mean ± standard deviation. *P* ≤ 0.05 values or less were considered to indicate statistically significant difference.

## 3. Results

### 3.1. Antibacterial Susceptibility

**Figure 1:**
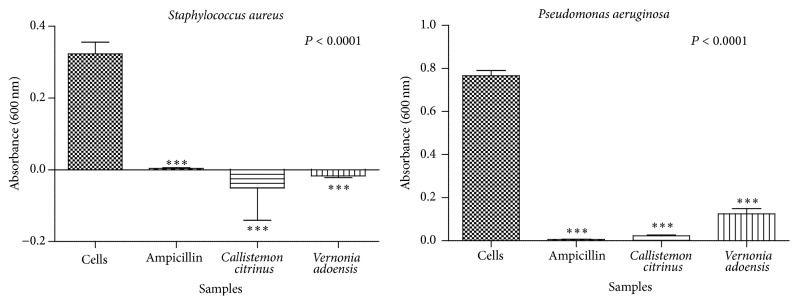
The effect of ampicillin,* C. citrinus*, and* V. adoensis* alkaloids on growth of* S. aureus* and* P. aeruginosa*. Concentrations of 1.67 mg/mL and 1 × 10^6^ cfu/mL of the extract and bacteria, respectively, were used. Values are expressed as mean absorbance at 600 nm wavelength ± standard deviation (*n* = 8).

The effects of* C. citrinus* and* V. adoensis* extracts on bacterial species were determined by measuring preincubation and postincubation absorbance readings at 600 nm. At an initial screening concentration of 1.67 mg/mL, both alkaloids from* C. citrinus* and* V. adoensis* inhibited bacterial growth significantly (*P* < 0.001, Figure 1).* C. citrinus* alkaloids were the most potent in inhibiting growth of* P. aeruginosa* with the mean absorbance for the cells exposed to this extract being 0.023 AU whilst that of* V. adoensis* alkaloids had 0.125 AU. Ampicillin as was expected almost killed all the cells in the positive controls as the recorded mean absorbance was 0.0062 AU. Generally,* P. aeruginosa* was less susceptible to the antibacterial effect of the alkaloids and ampicillin as compared to* S. aureus.*

### 3.2. Determination of MIC and MBCs

The MIC was identified for the well in which there was the least visible MTT color. The extracts from* C. citrinus* were the most potent with the lowest MIC of 0.025 mg/mL against* S. aureus* and 0.21 mg/mL against* P. aeruginosa.* The least potent extracts were those from* V. adoensis*, the MIC being 0.21 mg/mL against* S. aureus*.* C. citrinus* extracts only had a bactericidal action against* S. aureus* only with an MBC of 0.835 mg/mL (Table 1) whereas the MBC for ampicillin was 0.008 mg/mL [25]. Alkaloids from* C. citrinus* and* V. adoensis* had bacteriostatic effect against both bacterial species.

### 3.3. Drug Accumulation Assay

**Figure 2:**
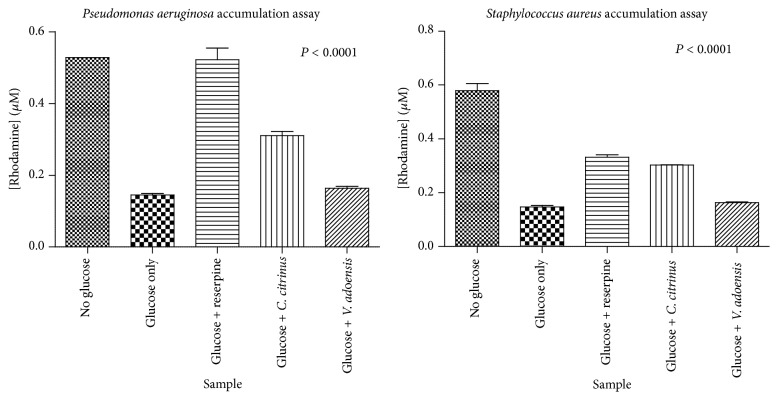
The accumulation of Rhodamine 6G over time. The graphs are a plot of Rhodamine 6G concentration inside the cells after 30 minutes of incubation against the sample used (plant alkaloid or reserpine). Glucose was used as positive control to provide a baseline increase in accumulation and as energy for the ATP-dependent efflux pumps. Error bars denote the standard deviations from the mean (*n* = 4).

The effects of the extracts on drug accumulation were determined using Rhodamine 6G, a fluorescent dye that is actively pumped out by the ATP-dependent efflux pumps of both species of bacteria in this study [26]. The highest accumulation of Rhodamine 6G was by* C. citrinus* alkaloids against* P. aeruginosa* which was 121% increase from the glucose only control.* S. aureus* was shown to be less susceptible to efflux pump inhibition as an increase in accumulation of 114% was obtained. For* V. adoensis,* less inhibitory activity was noted with only 14% increases in accumulation from the control. Thus,* C. citrinus* alkaloids inhibited efflux pumps to a greater extent as compared to* V. adoensis* alkaloids. Interestingly,* P. aeruginosa* proved to be more susceptible to efflux pump inhibition than* S. aureus* as the standard inhibitor reserpine's activity was more notable against* P. aeruginosa* than against* S. aureus* (Figure 2 and Table 2).

## 4. Discussion

The search for antimicrobial compounds for the benefit of humanity is necessitated by the inherent ability of pathogens to develop and adopt mechanisms of resistance against antibiotics. Potentially harmful side effects associated with use of new chemical entities synthesized artificially and the unsustainably high costs of drug development are slowly shifting the focus to plant derived phytochemicals of medicinal significance [27]. Coformulation of naturally sourced antimicrobial adjuvants like efflux pump inhibitors with old and new generation antibiotics remains a novel mechanism of countering antimicrobial resistance.

The effects* of C. citrinus* and* V. adoensis* extracts were determined by carrying out antibacterial susceptibility tests, minimum inhibitory concentration (MIC), and minimum bactericidal concentration (MBC) determinations. The antibacterial susceptibility tests showed that the alkaloid extract had potent antibacterial properties. Results from this study showed* S. aureus* to be more susceptible to both alkaloid extracts than* P. aeruginosa* which was expected as previous studies confirm that Gram-positive strains are more sensitive to malicious xenobiotics than Gram-negative bacteria [8].* V. adoensis* alkaloid extract also showed significant antibacterial activity second to* C. citrinus*. Similar margins of inhibition were obtained by Aliyu et al. [17] when chloroform fractions of* Vernonia* species were tested against methicillin resistant* S. aureus* (MRSA) and Gram-negative bacteria as well. Chloroform and ethanol extracts of* C. citrinus* were found to be active against both* S. aureus* and* P. aeruginosa* and other bacteria like* E. coli* and* S. typhi* via disc diffusion methods [28, 29]. Although the extracts used were crude, alkaloids have been found to be present in the chloroform and ethanol solvents of extraction [29]. In this regard, the alkaloid extract of* C. citrinus* and* V. adoensis* might have broad spectrum activity since they exhibited activity against both Gram-positive and Gram-negative bacteria.

The MIC and MBC assay confirmed that alkaloids form* C. citrinus* were more potent extracts against* S. aureus* which had an MIC value of 0.0025 mg/mL and an MBC of 0.835 mg/mL. The MBC of ampicillin was 0.008 mg/mL [25]; hence, the activity of the alkaloids is far outweighed by the standard antibiotic.* V. adoensis* alkaloids were least potent with MIC of 0.42 mg/mL and 0.21 mg/mL against* P. aeruginosa* and* S. aureus,* respectively. Bactericidal effects were shown by* C. citrinus* alkaloids against* S. aureus* only (Table 1). A bacteriostatic effect against* P. aeruginosa* by all plant alkaloid extracts could be due to the pathogen's thick outer membrane that is highly hydrophobic and possibly provided a permeability barrier to the extract [25]. This bacterium is generally less sensitive to antimicrobials [8]. In a previous study by Krishna et al. [29], chloroform extracts of alkaloids from* C. citrinus* were found to be inhibitors of growth in bacteria. MIC values obtained for the Gram-positive (*B. subtilis* and* B. pumilis*) and Gram-negative bacteria (*E. coli*) were less than that of streptomycin, the standard antibiotic used in that study.

ATP-dependent efflux pumps are one of the main mechanisms of preventing accumulation of effective concentrations of antibiotics at molecular target sites inside the bacterial cell. The major efflux pump systems studied in Gram-negative strains like* P. aeruginosa* are MexXY-OprM or MexCD-OprJ which have been associated with acquired multidrug resistance [15, 16]. In Gram-positive bacteria such as* S. aureus* transporters of the Major Facilitator superfamily (MFS), QacA, QacB, NorA, and NorB were found in strains causing hospital-acquired infections and conferring resistance to fluoroquinolones and puromycin [16]. Inhibition of efflux pumps is a plausible mechanism that can be employed to effectively combat the consequences of resistance. Inhibition of efflux pumps was also found to decrease the MICs for both antibiotic susceptible and resistant bacteria and reversed acquired resistance among selected* P. aeruginosa* strains [30]. The alkaloids extract from both plants was able to cause accumulation of Rhodamine 6G in the cells of* S. aureu*s and* P. aeruginosa.* However,* C. citrinus* alkaloids were potent inhibitors compared to alkaloids from* V. adoensis* and caused 141% and 121% increases in accumulation of R6G in* S. aureu*s and* P. aeruginosa*, respectively.* V. adoensis* alkaloid extracts only managed to make the cells accumulate only a 14% increase in the two bacterial species. Previous work on efflux pump activity revealed* V. adoensis* crude extracts to be more active in inhibiting* S. aureus* than* C. citrinus* [25]; in light of the results of this present study, such activity of* V. adoensis* might have been due to other phytoconstituents present in the crude extracts. The greatest accumulation of R6G was caused by exposure of cells to* C. citrinus* alkaloids against* P. aeruginosa.* These results are in agreement with the work of Chitemerere and Mukanganyama [25] in which* C. citrinus* crude extracts caused the most significant accumulation in* P. aeruginosa* as well. Therefore,* P. aeruginosa* was more sensitive to efflux pump inhibition by phytoconstituents than Gram-positive microbes, although this bacterium was less sensitive to growth inhibition. This is an unexpected finding which was also observed with crude extracts that exhibited significant inhibition of 64–100% of drug accumulation of Rhodamine 6G in* P. aeruginosa* cell [25].

It is important to identify the agents that block efflux of drugs from within the bacteria as they are potential sources of adjuvants in coformulations with conventional antibiotics [31]. A clinical example is the successful coformulation of clavulanic acid, a penicillinase inhibitor, with amoxicillin, an old conventional beta-lactam antibiotic in the coamoxiclav generic.* In vitro* studies of synergistic effects of combinations of an MDR pump inhibitor and other antimicrobial phytoconstituents as well as conventional antibiotics have been noted with significant results [8]. Previous studies have already proved the effectiveness of* C. citrinus* extract for* in vitro* antibacterial activity [28, 29, 32].

In conclusion, the alkaloid extracts from* C. citrinus* and* V. adoensis* have shown some antibacterial activity on* S. aureus* and* P. aeruginosa*. The* C. citrinus* alkaloid extract showed more potent growth inhibitory activity than that from* V. adoensis* with some bactericidal effects. However, activity from the extracts was far much less compared to ampicillin.* P. aeruginosa* was most susceptible to efflux pump inhibition by alkaloids extracted from* C. citrinus* which also caused notable accumulation in* S. aureus*. The demonstration of* P. aeruginosa* as being more susceptible to efflux pump inhibition means that formulations of medicinal products with EPI activity may have potential acceptable efficacy against this bacterial species. Further work needs to be done to isolate the specific chemicals that have antibacterial activity.

## Figures and Tables

**Table 1:** A summary table for the minimum inhibitory concentration (MIC) and minimum bactericidal concentration (MIC) assays.

Bacteria	G+/G−	Alkaloid extract	MIC (mg/mL)	MBC (mg/mL)
*S. aureus*	G+	*Callistemon citrinus*	0.0025	0.835
		*Vernonia adoensis*	0.21	—

*P. aeruginosa*	G−	*Callistemon citrinus*	0.21	—
		*Vernonia adoensis*	0.42	—

**Table 2:** A summary table for the concentration of R6G that accumulated in the bacterial cells after exposure to the extracts.

Bacteria	Glucose	Reserpine	*C. citrinus*	*V. adoensis*
*S. aureus*	0.14 ± 0.004	0.33 ± 0.008 (135%)	0.30 ± 0.0006 (114%)	0.16 ± 0.002 (14%)

*P. aeruginosa*	0.14 ± 0.003	0.52 ± 0.03 (271%)	0.31 ± 0.01 (121%)	0.16 ± 0.005 (14%)

Values are expressed as mean (*µ*M) ± standard deviation (*n* = 4) and values in parenthesis represent the percentages increases in accumulation from the control (glucose only).

## Abbreviations

CFU: Colony forming units
DMSO: Dimethyl sulfoxide
MIC: Minimum inhibitory concentration
MBC: Minimum bactericidal concentration.
